# Diagnostic Performance of Angiography-Derived Quantitative Flow Ratio: A Systematic Review and Meta-Analysis

**DOI:** 10.3390/medsci14010051

**Published:** 2026-01-19

**Authors:** Guo Huang, Pu Ge, He Zhu, Sheng Han, Luwen Shi

**Affiliations:** 1 Department of Pharmacy Administration and Clinical Pharmacy, School of Pharmaceutical Sciences, Peking University, Beijing 100191, China; 2 International Research Center for Medicinal Administration, Peking University, Beijing 100191, China; 3 School of Traditional Chinese Medicine, Beijing University of Chinese Medicine, Beijing 100029, China

**Keywords:** quantitative flow ratio, fractional flow reserve, angiography, significant stenosis

## Abstract

**Background:** Quantitative flow ratio (QFR) is a novel technology to assess the functional significance of coronary stenoses based on standard coronary angiography, which can be alternatives to invasive fractional flow reserve (FFR) assessment. However, the evidence is limited to single-center studies and small sample sizes. This study systematically determined the diagnostic performance of QFR to diagnose functionally significant stenosis with FFR as the reference standard. **Methods:** A systematic review and meta-analysis of studies assessing the diagnostic performance of angiography-derived QFR systems were performed. All relevant studies from six literature databases were searched and screened according to the inclusion and exclusion criteria. The pooled sensitivity, specificity, positive likelihood ratio (LR+), negative likelihood ratio (LR−), and diagnostic odds ratio (DOR), along with their 95% confidence intervals (CIs), were calculated using DerSimonian–Laird methodology. The summary receiver operating characteristic (SROC) curve and area under the curve were estimated. Meta-regression analysis was performed to identify a potential source of heterogeneity. **Results:** Fifty-seven studies comprising 13,215 patients and 16,125 vessels were included in the final analysis. At the vessel level, the pooled sensitivity and specificity of QFR for detecting a significant coronary stenosis were 0.826 (95% CI: 0.798–0.851) and 0.919 (95% CI: 0.902–0.933). Pooled LR+ and LR− were 10.198 (95% CI: 8.469–12.281) and 0.189 (95% CI: 0.163–0.219), with a pooled DOR of 53.968 (95% CI: 42.888–67.910). The SROC revealed an area under the curve (AUC) of 0.94 (95% CI: 0.91–0.96). The summary AUCs were 0.90 (95% CI: 0.87–0.92) for fixed-flow QFR (fQFR), 0.95 (95% CI: 0.92–0.96) for contrast-flow QFR (cQFR), 0.97 (95% CI: 0.95–0.98) for Murray law-based QFR (μQFR), and 0.91 (95% CI: 0.89–0.94) for non-specified QFR. The adjusted pooled DORs were as follows: 126.25 for μQFR, 45.49 for cQFR, 26.12 for adenosine-flow QFR (aQFR), 25.88 for fQFR, and 36.54 for non-specified QFR. **Conclusions:** The accuracy of angiography-derived QFR was strong to assess the functional significance of coronary stenoses with FFR as a reference. μQFR demonstrated the highest diagnostic performance among the five evaluated modes.

## 1. Introduction

Coronary artery disease (CAD), primarily caused by atherosclerosis-induced narrowing or occlusion of the coronary arteries, is one of the most prevalent cardiovascular diseases globally [1]. In fact, CAD is the leading cause of death worldwide, accounting for approximately 16% of global mortality [2]. Accurate assessment of CAD, particularly in cases of intermediate coronary artery stenosis, is crucial for evaluating myocardial ischemia and determining the appropriate next steps in treatment. Coronary angiography (CAG) is the established standard for identifying CAD. However, angiographic images often fall short in determining the functional significance of a stenosis. This limitation can result in unnecessary revascularizations or, conversely, the deferral of necessary interventions [3,4].

Pressure-derived fractional flow reserve (FFR) is widely regarded as the gold standard for evaluating the functional significance of coronary lesions and detecting lesion-specific ischemia; it is strongly endorsed by clinical guidelines as a crucial tool for guiding revascularization decisions [5,6]. FFR is defined as the ratio of maximal myocardial blood flow in the presence of an epicardial stenosis to that in a disease-free vessel, reflecting the fraction of normal blood flow supplied by the affected vessel [7]. An FFR value ≤ 0.80 indicates a functionally significant stenosis, where revascularization has been shown to provide better outcomes compared to conservative treatment [8]. FFR is highly sensitive and specific in detecting myocardial ischemia [9], and studies consistently demonstrate that FFR-guided percutaneous coronary intervention (PCI) reduces the number of implanted stents and significantly improves clinical outcomes compared to angiography alone [10,11]. However, the clinical adoption of FFR remains limited due to high costs, prolonged procedure time, hyperemia-induced discomfort, and risks such as complications and coronary dissection [12]. Challenges also include difficulty navigating pressure wires, reliance on angiographic assessments, and insufficient reimbursement support [13,14].

To enhance access to functional lesion assessment during invasive coronary angiography, quantitative flow ratio (QFR), as a wire-free FFR rapid analysis system, was recently developed [15]. This approach utilizes artificial intelligence to reconstruct three-dimensional vascular models from coronary angiography images and simulate the calculation of FFR indicators based on thrombolysis in myocardial infarction (TIMI) frame counting [16]. Compared to FFR, QFR eliminates the need for invasive physiological measurements, pharmacological hyperemia, and additional cost; meanwhile, it offers a shorter computation time, making it a more efficient alternative [17]. QFR can be derived using four distinct flow models: (1) fixed-flow QFR (fQFR), which uses a fixed empiric hyperemic flow velocity (HFV), based on previous FFR studies; (2) adenosine-flow QFR (aQFR), using the measured HFV obtained from coronary angiography during adenosine-induced maximal hyperemia; (3) contrast-flow QFR (cQFR), with modeled HFV derived from coronary angiography performed without pharmacologically induced hyperemia [18]; (4) Murray law-based QFR (μQFR), which enables automatic contour delineation and swift FFR simulation using a single CAG image from a specific angle [19]. QFR has been extensively investigated and has exhibited robust diagnostic features in European, Asian, and US populations [20]. Moreover, recent studies have verified good correlation and agreement between QFR and FFR, highlighting its potential clinical utility [21,22].

Given the intensive ongoing research in this field and the continuous evolution of these systems, interventionalists require a comprehensive understanding of the diagnostic capabilities of two distinct technologies. However, previous meta-analyses may have been underpowered due to their limited size, and there exists no systematic comparison between various QFR modes and FFR in assessing diagnostic performance [23,24]. To address this gap and enhance our understanding of QFR computation, we conducted a systematic review and quantitative meta-analysis. Our objective was to update the available information by comparing the fQFR, aQFR, cQFR, and μQFR flow models with invasive FFR in evaluating the functional significance of coronary stenoses.

## 2. Methods

### 2.1. Data Sources and Searches

PubMed, EMBASE, the Cochrane Library, the China National Knowledge Infrastructure (CNKI), the Wanfang Data Knowledge Service Platform (WANFANG data), and the China Biomedicine Database (Sinomed) were systematically searched from their inception to 10 November 2024 for published studies in both English and Chinese, using the terms “quantitative flow ratio or QFR” and “fractional flow reserve or FFR”.

Additionally, a manual reference check of literature was performed for eligible papers, and two independent reviewers examined the references to exclude duplicate or overlapping data. The eligibility of the articles, the data extraction, and quality assessment were independently evaluated by two reviewers, with a third review consulted to resolve any disagreements. Articles containing original material were retrieved and assessed in detail, and the references cited within these publications were further reviewed to identify additional relevant studies.

### 2.2. Study Selection

Inclusion criteria included the following: (1) the diagnostic performance of QFR was assessed using FFR as the standard reference, with the FFR threshold to diagnose coronary stenosis severity set at ≤0.80; (2) sufficient data must be provided in the full text to derive the number of true positives (TPs), false negatives (FPs), false positives (FPs), and true negatives (TNs), which construct the 2 × 2 contingency table. Exclusion criteria included the following: (1) case reports, abstracts, reviews, posters, comments, animal experiments, or other non-original articles; (2) duplicate publications or studies with overlapping sample data; and (3) articles not published in Chinese or English.

### 2.3. Data Extraction and Quality Assessment

The following information from the included studies was extracted: first author, publication year, study design, baseline characteristics of patients and lesions, clinical presentation, cutoffs of QFR and FFR, QFR measurements, and diagnostic performances. If a study compared multiple QFRs, each single QFR was analyzed separately. We prespecified the analyses according to five subgroups: (1) fQFR vs. FFR; (2) aQFR vs. FFR; (3) cQFR vs. FFR; (4) μQFR vs. FFR; (5) non-specified QFR vs. FFR.

Study quality was assessed by the second version of Quality Assessment of Diagnostic Accuracy Studies (QUADAS-2) scale [25]. Discrepancies between reviewers were judged by a third person. This review was performed in accordance to the PRISMA (Preferred Reporting Items for Systematic Reviews and Meta-Analyses) guidelines. This systematic review and meta-analysis were registered in PROPERO (CRD 42023489289). The study was approved by the ISB of the School of Public Health, Fudan University (IRB# 2019-07-0767).

### 2.4. Statistical Analysis

This was a study-level meta-analysis based on aggregated data extracted from included studies, with the primary analysis conducted at the per-vessel level. All variables were presented as numbers and percentages (%), mean ± standard deviation (SD), or medians [interquartile range (IQR)] as appropriate. On the basis of the results from the 2 × 2 tables, the pooled sensitivity, specificity, positive likelihood ratio (LR+), negative likelihood ratio (LR−), and diagnostic odds ratio (DOR), along with their 95% confidence intervals (CIs), were calculated using DerSimonian–Laird methodology [26] to assess the diagnostic performance of QFR for diagnosing significant coronary stenosis. The DOR, calculated as LR+/LR−, reflects the ability of a test to distinguish, in this case, functionally and non-functionally significant lesions. A higher DOR indicates better diagnostic performance of the system. The summary receiver operating characteristic (SROC) curve was also calculated, in which we drew all the points of sensitivity and 1-specificity and adjusted the weighted regression curve using Moses’ Model [27]. The area under the SROC curve (AUC) serves as a global measure of test performance: excellent detection (AUC range of 0.90–1.00), good detection (AUC range of 0.80–0.90), fair detection (AUC range of 0.70–0.80), poor detection (AUC range of 0.60–0.70), and failure (AUC range of 0.50–0.60) [28].

Cochran’s Q test and the measured inconsistency (I^2^) index were calculated to assess potential heterogeneity across studies. Studies with *p* < 0.05 or I^2^ > 50% were defined as significantly heterogeneous. Data with heterogeneity were pooled using a DerSimonian–Laird random-effects model [26], whereas the Mantel–Haenszel fixed-effects model was adopted if there was no significant heterogeneity. Meta-regression analysis was performed to identify a potential source of heterogeneity. A multilevel linear regression model (method = REML, weight = 1/variance of odds) was used at the per-vessel level to explore the effects of the QFR modes on the pooled DOR while controlling for other effects [number of vessels (less or more than 200), year of publication (before or after 2020), country (Asian or others), study design (prospective or retrospective), and research type (single-center or multicenter)]. Using the above model, the adjusted pooled DORs of QFR modes were calculated and graphed.

In addition, Deek’s funnel plot asymmetry test was employed to investigate publication bias, and *p* < 0.05 indicated a significant asymmetry. All *p* values were two-tailed, with statistical significance set at *p* < 0.05. Analyses were performed using Review Manager 5.3, Stata/SE 12.0, and SAS 9.4.

## 3. Results

### 3.1. Literature Search

**Figure 1:**
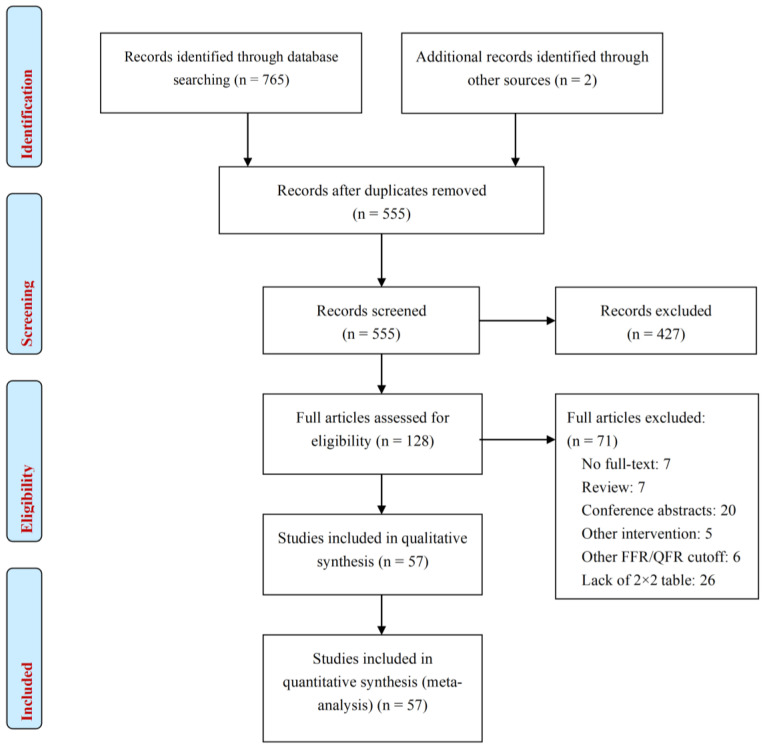
Literature search of eligible studies.

A total of 765 records were initially detected with the terms used. After removal of duplicates and screening by title and abstract, 128 full articles received a complete review. Of those, 57 QFR studies met the inclusion criteria and were used for the qualitative and quantitative meta-analysis (Figure 1) [17,19,21,22,29,30,31,32,33,34,35,36,37,38,39,40,41,42,43,44,45,46,47,48,49,50,51,52,53,54,55,56,57,58,59,60,61,62,63,64,65,66,67,68,69,70,71,72,73,74,75,76,77,78,79,80,81].

### 3.2. Study Characteristics

A total of 57 studies evaluated QFR, classified as follows: 11 studies on fQFR (2403 patients, 2958 vessels), 33 studies on cQFR (6009 patients, 7830 vessels), 2 studies on aQFR (90 patients, 99 vessels), 9 studies on μQFR (3108 patients, 3378 vessels), and 8 studies on non-specified QFR (1605 patients, 1860 vessels). Notably, some studies included more than one QFR mode [21,29,30,31,32,33,36,37,72].

The details of the included studies, such as year of publication, country, study design, research type, standard reference, FFR cutoff, and QFR cutoff, are described in Table 1. Baseline characteristics of patients, including demographics, clinical symptoms, cardiovascular risk factors, and cardiovascular history, are summarized in the Supplementary Materials online, Tables S1 and S2. The characteristics of target vessels are detailed in the Supplementary Materials online, Table S3. Individual study estimates of per-vessel diagnostic accuracy of QFR for identifying the functional significance of coronary stenoses are presented in the Supplementary Materials online, Table S4. The accuracy ranged from 61.54% to 98.3%, sensitivity ranged from 40.00% to 100.00%, specificity ranged from 27% to 100.00%, and AUC ranged from 0.821 to 0.987.

### 3.3. Pooled Diagnostic Performance

**Figure 2:**
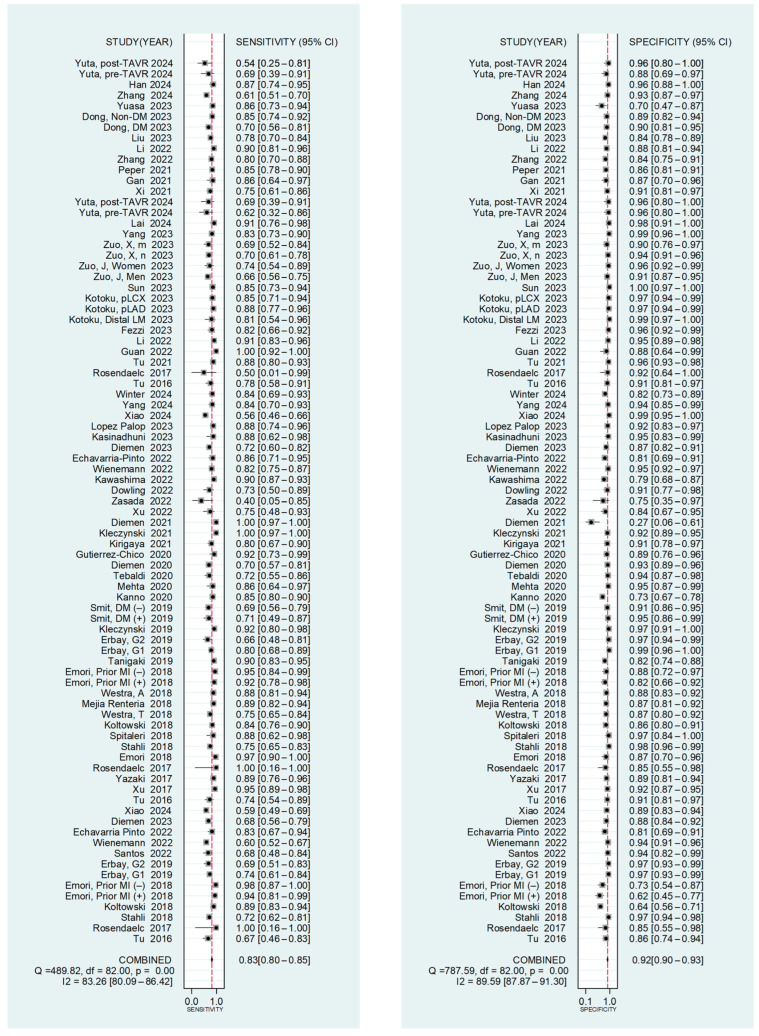
Forest plots for sensitivity and specificity of QFR.

**Figure 3:**
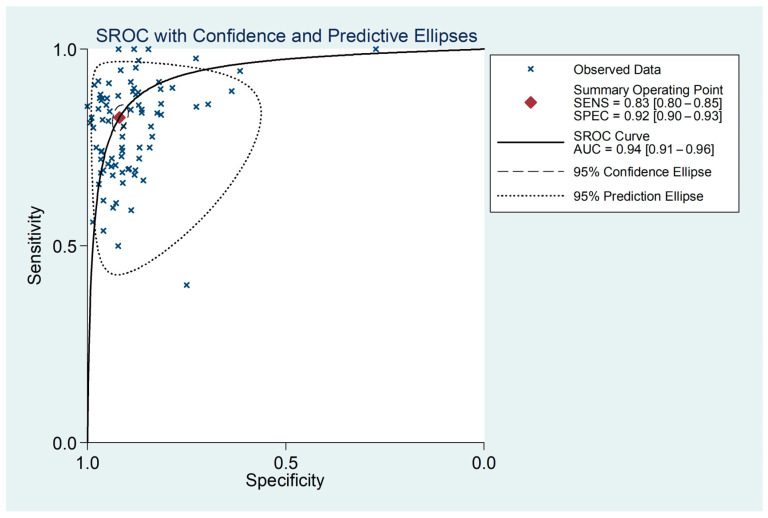
Summary receiver operating characteristic curve for QFR.

The pooled diagnostic performance for each QFR mode is summarized in Table 2. At the vessel level, the pooled sensitivity and specificity of QFR for detecting a significant coronary stenosis were 0.826 (95% CI: 0.798–0.851) and 0.919 (95% CI: 0.902–0.933) (Figure 2), respectively. Pooled LR+ and LR– were 10.198 (95% CI: 8.469–12.281) and 0.189 (95% CI: 0.163–0.219) (Supplementary Materials online, Figure S1), with a pooled DOR of 53.968 (95% CI:42.888–67.910) (Supplementary Materials online, Figure S2). The SROC revealed an area under the curve (AUC) of 0.94 (95% CI: 0.91–0.96) (Figure 3).

For individual modes, fQFR exhibited a sensitivity of 0.775 (95% CI: 0.685–0.845) and specificity of 0.886 (95% CI: 0.817–0.931), cQFR had a sensitivity of 0.854 (95% CI: 0.814–0.887) and specificity of 0.908 (95% CI: 0.882–0.930), and μQFR demonstrated a sensitivity of 0.829 (95% CI: 0.775–0.873) and specificity of 0.967 (95% CI: 0.952–0.977). For studies that did not specify the mode of QFR, the sensitivity was 0.790 (95% CI: 0.735–0.837) and the specificity was 0.883 (95% CI: 0.855–0.906) (Table 2). However, data on the diagnostic accuracy of aQFR compared with FFR were limited to only two studies. Owing to variable reporting and substantial heterogeneity in results across these studies, a full meta-analysis was not feasible. Meta-analyses of DORs and AUCs were also conducted for specific QFR modes. The DORs were as follows: 26.766 (95% CI: 17.645–40.603) for fQFR, 58.191 (95% CI: 42.801–79.116) for cQFR, 142.051 (95% CI: 76.295–264.480) for μQFR, and 28.396 (95% CI: 20.795–38.775) for non-specified QFR. The summary AUCs were 0.90 (95% CI: 0.87–0.92) for fQFR, 0.95 (95% CI: 0.92–0.96) for cQFR, 0.97 (95% CI: 0.95–0.98) for μQFR, and 0.91 (95% CI: 0.89–0.94) for non-specified QFR (Table 2).

### 3.4. Meta-Regression Analysis

**Figure 4:**
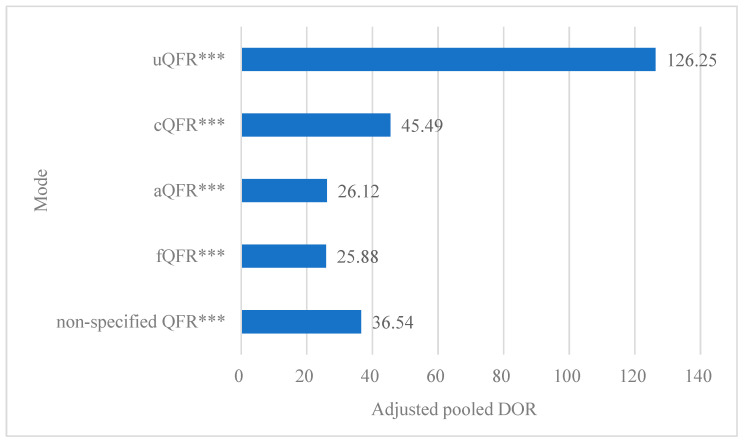
Adjusted pooled diagnostic odds ratio of QFR. “***” indicates that the adjusted pooled DOR for the mode was significantly higher than 1, with *p* < 0.001.

The results revealed significant differences in the log values of pooled DORs among the QFR modes. While the log (pooled DOR) for the cQFR mode showed no significant differences compared to other QFR modes, it was notably lower than that of μQFR and higher than that of fQFR (Table 3). The adjusted pooled DORs, calculated using the specified modes, were as follows: 126.25 for μQFR, 45.49 for cQFR, 26.12 for aQFR, 25.88 for fQFR, and 36.54 for non-specified QFR. Importantly, all the adjusted pooled DORs were significantly higher than 1 (Figure 4).

### 3.5. Study Quality and Publication Bias

The quality of QFR studies was summarized in the Supplementary Materials online, Figure S3. Nearly all studies demonstrated a low risk of bias in the index test and reference standard. Nevertheless, 12% (7/57) of the studies had a high risk of bias in patient selection, primarily due to the lack of consecutive inclusion of patients. Additionally, six studies exhibited a high risk of bias in flow and timing, as not all samples were included in the final analysis. No significant publication bias was detected according to Deek’s funnel plot asymmetry test, with a bias coefficient of 1.855 (*p* = 0.679).

## 4. Discussion

The diagnostic accuracy of QFR has been extensively investigated, with 57 studies included in this review, encompassing a total of 13,215 patients and 16,125 vessels. Our meta-analysis confirms that QFR demonstrates strong diagnostic performance, with a pooled AUC of 0.94 (95% CI: 0.91–0.96). Additionally, the high pooled sensitivity [0.826 (95% CI: 0.798–0.851)] and specificity [0.919 (95% CI: 0.902–0.933)] further validate the reliability of this novel tool in assessing coronary stenosis. The strong pooled LR+ [10.198 (95% CI 8.469–12.281)] and low pooled LR− [0.189 (95% CI 0.163–0.219)] provide compelling diagnostic evidence of the usefulness of QFR in the clinical setting. Moreover, the high pooled DOR suggests that a positive QFR lesion is 54 times more likely to correspond to a functionally significant lesion (measured FFR ≤ 0.80) compared to a non-functionally significant lesion.

QFR stands as a pioneering wire- and adenosine-free FFR rapid analysis system, offering a multitude of advantages: (1) Non-invasive procedure: by leveraging angiographic images, QFR eliminates the necessity for invasive procedures, thereby minimizing patient risk and discomfort. (2) Accuracy and reliability: QFR harnesses advanced artificial intelligence algorithms to enhance the precision and automation of image processing and blood flow simulation [82]. This reduces the influence of human error and subjective judgment, ultimately improving the accuracy and reliability of computational hemodynamic assessments. (3) Clinical effectiveness: QFR has undergone rigorous validation in numerous clinical trials, demonstrating its consistency and correlation with traditional wire-based methods. Furthermore, its guidance during PCI treatment has facilitated better patient selection, leading to improved patient outcomes [64,83]. With a positive primary endpoint in FAVOR III China, demonstrating increasing benefits up to 2 years [84], alongside procedural advantages such as lower costs compared to FFR, the path is paved for broader adoption of functional lesion evaluation. (4) Rapid computational analysis [17,41]: the technology’s capacity to swiftly process static images streamlines the diagnostic workflow, significantly reducing the time required in clinical practice. These attributes position QFR as a superior alternative in the realm of functional lesion evaluation.

More specifically, the summary AUCs for fQFR, cQFR, μQFR, and non-specified QFR were 0.90 (95%CI 0.87–0.92), 0.95 (95%CI 0.92–0.96), 0.97 (95%CI 0.95–0.98), and 0.91 (95%CI 0.89–0.94), respectively. These findings underscore the strong diagnostic performance of QFR across its various modes. Our meta-regression revealed that the log value of the pooled DOR for cQFR was nearly double that of fQFR. Similar outcomes were presented in smaller prospective observational studies by Tu et al. [29], van Rosendael et al. [30], and Echavarría-Pinto et al. [36], supporting the robustness of these observations. They demonstrated that the QFR computation, based on a patient-specific contrast-flow model derived from coronary angiography without pharmacologic hyperemia induction, achieved superior diagnostic accuracy compared to the fixed-flow approach. The cQFR employs frame count analysis from regular (non-hyperaemic) angiographic projections to simulate hyperemic flow velocity. Conversely, the fQFR applies a fixed empiric hyperemic flow velocity derived from prior FFR studies, thereby eliminating the requirement for TIMI frame counting and disregarding the impact of coronary microvasculature circulation. Consequently, the diagnostic accuracy of fQFR was found to be inferior to that of cQFR in the present study.

This study further revealed that μQFR exhibited superior diagnostic accuracy through the utilization of QFR computations based on a single angiographic view, thereby eliminating the necessity for 3D angiographic reconstruction. Several factors may account for this outcome. Firstly, the μQFR computation precisely delineated the lumen contours of side branches and reconstructed a step-down reference diameter function for the hypothetical healthy vessel, as opposed to assuming a linear tapering of the reference vessel size. This methodology captured the inherent fractal physiology of bifurcations, facilitating a more accurate quantification of lesion severity, which is a pivotal determinant of FFR. Secondly, the μQFR computation leveraged angiographic views with optimal image quality. Adequate exposure of the stenotic segment enhanced the precision in quantifying the geometry of the vessel under investigation. Notably, μQFR based on a single angiographic view was reported to possess technical advantages, including easier operation, shorter analysis time, and enhanced reproducibility [17,19,85].

Despite its demonstrated diagnostic accuracy, QFR possesses inherent limitations, particularly when considered within the broader context of comprehensive invasive coronary physiology assessment. Unlike invasive wire-based techniques such as coronary flow reserve (CFR) or acetylcholine provocation testing, QFR is fundamentally a computation derived from angiographic anatomy and contrast flow timing. Consequently, it cannot assess microvascular dysfunction or coronary vasospasm, which are crucial components of ischemic heart disease that require dedicated invasive diagnostics [43]. Furthermore, a key practical disadvantage is its lack of seamless integration into the interventional workflow. With wire-based methods, the same device used for physiological measurement can be immediately used to guide PCI if needed, whereas QFR, as an offline analytical tool, does not offer this “one-wire” procedural continuity. Its accuracy remains highly dependent on optimal angiographic image quality, as technical factors such as suboptimal lesion visualization, inadequate contrast filling, or limited projection angles can significantly impact computation reliability [53]. Additionally, the performance of QFR in complex anatomical scenarios (e.g., ostial or bifurcation lesions, severely tortuous vessels, diffuse disease) and in patients with specific clinical conditions (e.g., microvascular dysfunction, arrhythmias) requires further validation [86]. Future studies are warranted to address these technical and clinical boundaries, and head-to-head comparisons with other angiography-derived fractional flow reserve modalities may help refine its optimal use in real-world practice.

## 5. Limitations

Several limitations of our study merit consideration. Firstly, despite substantial evidence supporting the diagnostic accuracy of QFR assessment, the impact of specific patient or lesion characteristics (such as a history of recent myocardial infarction or coronary artery bypass grafting, multi-vessel disease, or the quality of angiographic images) on the diagnostic accuracy or clinical effectiveness of QFR remains largely unknown. Notably, the quality of angiographic material is of foremost importance for accurate QFR computation. Hence, further research is warranted to investigate parameters for automatically assessing the quality of angiographic acquisitions intended for QFR analysis. Secondly, the absence of individual patient-level data hindered deeper analysis to identify predictors of QFR accuracy. Thirdly, potential methodological and selection biases should be acknowledged. Despite employing two independent reviewers for study selection and data extraction and including both English and Chinese publications, language bias may still exist due to the exclusion of studies published in other languages. Fourth, the inconsistent reporting of specific patient and lesion characteristics—such as chronic total occlusion, valvular disease, or heart failure—precluded subgroup adjustment. This clinical heterogeneity likely contributed to residual statistical heterogeneity and limits the granularity of our conclusions. Consequently, well-designed, large-scale, multicenter prospective clinical trials are essential to better understand the role of FFR and QFR-guided assessments in complex clinical scenarios.

## 6. Conclusions

In conclusion, our analysis confirms the impressive diagnostic performance of QFR in detecting functional ischemia of coronary arteries, with pressure-wire measured FFR serving as the reference standard. Notably, μQFR demonstrated the highest diagnostic performance among the evaluated modes. QFR might be considered a reliable and useful alternative to pressure wire-based FFR due to its simplicity and non-invasive modality. However, this superiority should be interpreted with caution, given the observed heterogeneity, the lack of automated quality assessment for angiographic acquisitions, and the complexities inherent in clinical practice. Therefore, further randomized trials are warranted to unveil the value of a QFR-based strategy in patients requiring functional evaluation.

## Figures and Tables

**Table 1:** Study characteristics.

Characteristics	No.	Percent (%)	Characteristics	No.	Percent (%)
Year of publication			Study design		
2016	1	1.75	Prospective	20	35.09
2017	3	5.26	Retrospective	37	64.91
2018	8	14.04	Total	57	100.00
2019	4	7.02	Research type		
2020	5	8.77	Single-center	41	71.93
2021	7	12.28	Multicenter	16	28.07
2022	10	17.54	Total	57	100.00
2023	12	21.05	Standard reference		
Total	57	100.00	FFR	57	100.00
Country			Total	57	100.00
China	20	35.09	FFR cutoff		
Netherlands	7	12.28	≤0.08	57	100.00
Japan	7	12.28	Total	57	100.00
Poland	4	7.02	QFR cutoff		
Spain	4	7.02	≤0.80	57	100.00
Germany	3	5.26	Total	57	100.00
Italy	3	5.26			
Others	9	15.79			
Total	57	100.00			

**Table 2:** Meta-analysis of QFR.

Mode	Pooled Sensitivity	Pooled Specificity	Pooled LR+	Pooled LR−	Pooled DOR	AUC
QFR	0.826 (0.798–0.851)	0.919 (0.902–0.933)	10.198 (8.469–12.281)	0.189 (0.163–0.219)	53.968 (42.888–67.910)	0.94 (0.91–0.96)
fQFR	0.775 (0.685–0.845)	0.886 (0.817–0.931)	6.797 (4.440–10.407)	0.254 (0.185–0.349)	26.766 (17.645–40.603)	0.90 (0.87–0.92)
cQFR	0.854 (0.814–0.887)	0.908 (0.882–0.930)	9.334 (7.310–11.919)	0.160 (0.126–0.204)	58.191 (42.801–79.116)	0.95 (0.92–0.96)
μQFR	0.829 (0.775–0.873)	0.967 (0.952–0.977)	25.078 (16.810–37.412)	0.177 (0.132–0.237)	142.051 (76.295–264.480)	0.97 (0.95–0.98)
non–specified QFR	0.790 (0.735–0.837)	0.883 (0.855–0.906)	6.745 (5.517–8.247)	0.238 (0.188–0.300)	28.396 (20.795–38.775)	0.91 (0.89–0.94)

**Table 3:** Meta-regression of the log (pooled DOR) of QFR.

Variable	Estimate (95% CI)	Standard Error	*p* Value
Intercept	4.5176 (3.8247, 5.2104)	0.3477	<0.0001
Mode (control = cQFR)			
fQFR (1: yes, 0: no)	−0.5639 (−1.1147, −0.0131)	0.2764	0.0449
aQFR (1: yes, 0: no)	−0.5547 (−2.2753, 1.1660)	0.8634	0.5226
μQFR (1: yes, 0: no)	1.0209 (0.3377, 1.7042)	0.3428	0.0039
non-specified QFR (1: yes, 0: no)	−0.2191 (−0.8893, 0.4512)	0.3363	0.5168
No. of vessels (1: ≥200, 0: <200)	−0.1979 (−0.6380, 0.2423)	0.2208	0.3732
Publication year after 2020 (1: yes, 0: no)	−0.4868 (−0.9944, 0.0208)	0.2547	0.0599
Asian countries (1: yes, 0: no)	−0.2181 (−0.6670, 0.2309)	0.2253	0.3362
Design (1: prospective, 0: retrospective)	−0.2241 (−0.7402, 0.2919)	0.2589	0.3895
Single-center (1: yes, 0: no)	−0.2095 (−0.6769, 0.2579)	0.2345	0.3746

## Data Availability

The original contributions presented in this study are included in the article/Supplementary Material. Further inquiries can be directed to the corresponding author.

## Supplementary Materials

The following supporting information can be downloaded at: https://www.mdpi.com/article/10.3390/medsci14010051/s1, Figure S1: Forest plots of LR+ and LR- of QFR; Figure S2: Forest plots of DOR of QFR; Figure S3: Quality assessment of the included studies; Table S1: Demographics and clinical symptoms of patients in the studies; Table S2: Cardiovascular risk factors and history of patients in the studies; Table S3: Target vessel characteristics in the studies; Table S4: Individual study estimates of per-vessel diagnostic accuracy of QFR.
