# Supplementary material for: Diagnostic Performance of Angiography-Derived Quantitative Flow Ratio: A Systematic Review and Meta-Analysis
Source: Med Sci (Basel). 2026 Jan 19;14(1):51. doi: 10.3390/medsci14010051 (PMC12922042; doi:10.3390/medsci14010051)
Supplement: Supplementary file 1 [file medsci-14-00051-s001.zip › medsci-4034364-supplementary.pdf]

Supplementary Material

Table S1 Demographics and clinical symptoms of patients in the studies.

Study	No. of patients	Demographics			Clinical symptoms		
		Age, year	Male	BMI, kg/m^2	Chronic stable angina	Acute coronary syndrome	Other symptoms
**Mode=fQFR**							
Tu 2016	73	65.8 ± 8.9	61 (83.5)	26.3 ± 6.3	–	–	–
Rosendaelc 2017	17	64 ± 11	12 (71)	27.7 ± 5.3	–	–	–
Stähli 2018	436	71.5 (63.0–77.0)	296 (67.9)	26.0 (23.9–29.2)	313 (71.8)	123 (28.2)	0 (0.0)
Kołtowski 2018	268	66.3 ± 9.98	193 (72)	–	268 (100.0)	0 (0.0)	0 (0.0)
Emori, Prior MI (+) 2018	75	69 ± 9	62 (83)	–	–	–	–
Emori, Prior MI (–) 2018	75	70 ± 9	54 (72)	–	–	–	–
Erbay, G1 2019	225	71.0 (63.0–77.5)	142 (63.1)	26.2 (24.1–28.7)	163 (72.4)	62 (27.6)	0 (0.0)
Erbay, G2 2019	221	72.0 (64.0–77.0)	154 (73.0)	26.0 (23.8–29.4)	154 (73.0)	57 (25.8)	0 (0.0)
Santos 2022	69	64.55 ± 12.15	38 (55.1)	27.8 ± 6.4	25 (36.3)	21 (30.4)	23 (33.3)
Wienemann 2022	544	69.5 (61.3–77.0)	392 (72.06)	27.7 ± 4.8	429 (78.9)	114 (21.0)	1 (0.2)
Echavarría-Pinto 2022	66	66.1 ± 8.8	47 (71.2)	–	37 (56.1)	29 (43.9)	0 (0.0)
Diemen 2023	166	63.1 ± 9.3	130 (78)	27.3 ± 4.2	–	–	–
Xiao 2024	168	58.50 ± 10.52	119 (70.8)	–	0 (0.0)	168 (100)	0 (0.0)
**Mode=cQFR**							
Tu 2016	73	65.8 ± 8.9	61 (83.5)	26.3 ± 6.3	–	–	–
Xu 2017	308	61.3 ± 10.4	227 (73.7)	25.2 ± 3.3	106 (34.4)	202 (65.6)	0 (0.0)
Yazaki 2017	142	72.5 ± 9.5	100 (70.4)	23.9 ± 3.2	141 (99.3)	1 (0.7)	0 (0.0)
Rosendaelc 2017	17	64 ± 11	12 (71)	27.7 ± 5.3	–	–	–

Emori 2018	100	70 ± 10	71 (71)	–	–	–	–
Stähli 2018	436	71.5 (63.0–77.0)	296 (67.9)	26.0 (23.9–29.2)	313 (71.8)	123 (28.2)	0 (0.0)
Spitaleri 2018	45	62 ± 11	36 (80)	28 ± 5	0 (0.0)	45 (100.0)	0 (0.0)
Kołtowski 2018	268	66.3 ± 9.98	193 (72)	–	268 (100.0)	0 (0.0)	0 (0.0)
Westra, T 2018	172	61 ± 8	116 (67)	27 ± 4	53 (30.8)	0 (0.0)	119 (69.2)
Mejía-Rentería 2018	248	64.2 ± 10.3	188 (76)	26.2 (24.6–29.0)	174 (70.2)	74 (29.8)	0 (0.0)
Westra, A 2018	272	67 ± 10	196 (72)	27 ± 5	258 (94.9)	6 (2)	8 (3)
Emori, Prior MI (+) 2018	75	69 ± 9	62 (83)	–	–	–	–
Emori, Prior MI (–) 2018	75	70 ± 9	54 (72)	–	–	–	–
Tanigaki 2019	152	69 ± 9	98 (64)	–	–	–	–
Erbay, G1 2019	225	71.0 (63.0–77.5)	142 (63.1)	26.2 (24.1–28.7)	163 (72.4)	62 (27.6)	0 (0.0)
Erbay, G2 2019	221	72.0 (64.0–77.0)	154 (73.0)	26.0 (23.8–29.4)	154 (73.0)	57 (25.8)	0 (0.0)
Kleczyński 2019	50	66.0 ± 9.3	36 (72)	–	50 (100.0)	0 (0.0)	0 (0.0)
Smit, DM (+) 2019	66	67 ± 9	47 (71)	–	–	–	–
Smit, DM (–) 2019	193	67 ± 9	134 (69)	–	–	–	–
Kanno 2020	504	66.9 ± 9.4	88 (17.46)	–	–	–	–
Mehta 2020	47	65.1 ± 10.1	29 (61.7)	–	47 (100.0)	0 (0.0)	0 (0.0)
Tebaldi 2020	116	70 (44–85)	87 (75)	30.4 (18.73–81.93)	0 (0.0)	116 (100.0)	0 (0.0)
Diemen 2020	169	58 ± 9	101 (60)	27.0 ± 3.6	–	–	–
Gutiérrez-Chico 2020	59	63.4 ± 10.1	50 (84.75)	28.1 (25.4–30.6)	43 (72.9)	16 (27.1)	0 (0.0)
Kirigaya 2021	77	70 ± 9	62 (81)	–	77 (100.0)	0 (0.0)	0 (0.0)
Kleczynski 2021	221	82.0 (74.0–88.0)	91 (41.18)	27.0 (23.9–30.0)	221 (100.0)	0 (0.0)	0 (0.0)
Diemen 2021	115	64.9 ± 9.9	89 (77)	26.7 ± 3.8	114 (99.1)	1 (1)	0 (0.0)
Xu 2022	48	61.89 ± 9.18	30 (62.50)	–	–	–	–
Zasada 2022	12	73.8 ± 7.5	6 (50)	–	–	–	–
Dowling 2022	35	75.5 ± 6.5	25 (71)	28.7 ± 7.1	–	–	–

Kawashima 2022	183	67.0 ± 8.9	159 (86.9)	26.4 ± 3.6	–	–	–
Wienemann 2022	544	69.5 (61.3–77.0)	392 (72.06)	27.7 ± 4.8	429 (78.9)	114 (21.0)	1 (0.2)
Echavarría-Pinto 2022	66	66.1 ± 8.8	47 (71.2)	–	37 (56.1)	29 (43.9)	0 (0.0)
Diemen 2023	166	63.1 ± 9.3	130 (78)	27.3 ± 4.2	–	–	–
Kasinadhuni 2023	53	62.4 ± 9.1	43 (81.1)	–	34 (64.2)	19 (35.8)	0 (0.0)
Lopez-Palop 2023	81	70 ± 9.6	62 (76.5)	28.0 ± 3.9	42 (51.9)	39 (48.1)	0 (0.0)
Xiao 2024	168	58.50±10.52	119(70.8)	–	0 (0.0)	168 (100)	0 (0.0)
Yang 2024	78	61.5 ± 9.1	63 (80.8)	25.7 ± 3.1	28 (35.9)	50 (64.1)	0 (0.0)
Winter 2024	129	73 ± 8	109 (84)	28 ± 4	89 (69)	40 (31)	0 (0.0)
**Mode=aQFR**							
Tu 2016	73	65.8 ± 8.9	61(83.5)	26.3 ± 6.3	–	–	–
Rosendaelc 2017	17	64 ± 11	12(71)	27.7 ± 5.3	–	–	–
**Mode=μQFR**							
Tu 2021	306	–	–	–	–	–	–
Guan 2022	54	72.3 ± 7.2	41 (75.9)	–	49 (90.7)	5 (9.3)	0 (0.0)
Li 2022	167	62.4 ± 9.3	127 (76.0)	25.4 ± 3.4	61 (36.5)	77	29 (17.4)
Fezzi 2023	123	83.0 (78.3–86)	57 (46.3)	25.2 (22.8–27.5)	–	–	–
Kotoku, Distal LM 2023	300	66.8 ± 8.9	265 (88.9)	26.9 ± 4.3	–	–	–
Kotoku, pLAD 2023	300	66.8 ± 8.9	265 (88.9)	26.9 ± 4.3	–	–	–
Kotoku, pLCX 2023	300	66.8 ± 8.9	265 (88.9)	26.9 ± 4.3	–	–	–
Sun 2023	163	61.85 ± 8.77	122 (74.85)	–	–	–	–
Zuo, J, Men 2023	302	65.3 ± 10.3	–	24.9 ± 3.4	254 (84.1)	48 (15.9)	0 (0.0)
Zuo, J, Women 2023	158	68.1 ± 9.2	–	24.9 ± 3.8	141 (89.2)	17 (10.8)	0 (0.0)
Zuo, X, n 2023	461	65.7 ± 10.1	307 (66.6)	25.2 ± 3.4	397 (86.1)	64 (13.9)	0 (0.0)
Zuo, X, m 2023	73	68.9 ± 8.8	52 (71.2)	23.8 ± 3.3	64 (87.7)	9 (12.3)	0 (0.0)
Yang 2023	261	62.25 ± 9.40	190 (72.80)	–	–	–	–

Lai 2024	90	62.63 ± 9.22	63 (70)	–	–	–	–
Yuta, pre-TAVR 2024	25	85.8 ± 3.8	11 (44.0)	21.5 ± 2.8	–	–	–
Yuta, post-TAVR 2024	25	85.8 ± 3.8	11 (44.0)	21.5 ± 2.8	–	–	–
**Mode=non-specified QFR**							
Xi 2021	110	62.09	76 (69.1)	–	–	–	–
Gan 2021	52	62.8 ± 8.65	40 (76.9)	24.65 ± 3.11	–	–	–
Peper 2021	289	64.1 ± 10.4	211 (73.0)	27.4 ± 4.1	–	–	–
Zhang 2022	148	59.5 ± 9.7	101 (68.24)	25.72 ± 3.09	96 (64.86)	32 (21.62)	20 (13.51)
Liu 2023	337	60.00 (52.00–67.00)	228 (67.66)	25.98 ± 3.44	20 (5.9)	317 (94.1)	0 (0.0)
Dong, DM, 2023	99	65.1 ± 8.9	63 (64)	26.41 ± 2.8	–	–	–
Dong, Non-DM, 2023	125	65.1 ± 9.4	75 (60)	25.50 ± 3.0	–	–	–
Yuasa 2023	67	66.94 ± 12.14	54 (80.1)	26.29 ± 3.12	45 (67.2)	22 (32.8)	0 (0.0)
Zhang 2024	236	64.48 ± 9.63	160 (67.8)	–	–	108 (45.76)	–
Han 2024	92	59.8 ± 9.2	56 (60.9)	23.9 ± 3.1	77 (83.7)	15 (16.3)	0 (0.0)
Yuta 2024	25	85.8 ± 3.8	11 (44.0)	21.5 ± 2.8	–	–	–
Yuta 2024	25	85.8 ± 3.8	11 (44.0)	21.5 ± 2.8	–	–	–

Values are n (%), mean ± standard deviation or median (interquartile range). BMI, body mass index. G1, Group 1 (Diameter≤2.8mm); G2, Group 2 (Diameter > 2.8mm); DM, diabetes mellitus; LM, left main; pLAD, proximal LAD 10 mm distal to the LM bifurcation point; Plcx, proximal LCX 10 mm distal to the LM bifurcation point; TAVR, transcatheter aortic valve replacement.

Table S2 Cardiovascular risk factors and history of patients in the studies.

Study	Cardiovascular risk factors					Cardiovascular history		
	Smoking	Hypertension	Dyslipidemia	Diabetes	Family history of CAD	Prior MI	Prior PCI	Prior CABG
**Mode=fQFR**								
Tu 2016	–	32 (43.8)	–	17 (27.4)	–	23 (31.5)	28 (38.4)	2 (2.7)
Rosendaelc 2017	3 (18)	11 (65)	–	1 (6)	2 (12)	1 (6)	4 (24)	–
Stähli 2018	148 (34)	383 (87.8)	345 (79.1)	98 (22.5)	62 (14.2)	143 (32.8)	239 (54.8)	11 (2.5)
Kołtowski 2018	28 (10.4)	203 (75.7)	–	75 (28)	28 (10.4)	128 (47.8)	158 (59)	7 (2.6)
Emori, Prior MI (+) 2018	17 (23)	61 (81)	45 (60)	34 (45)	13 (17)	–	–	–
Emori, Prior MI (–) 2018	23 (31)	64 (85)	46 (61)	36 (48)	12 (16)	–	–	–
Erbay, G1 2019	–	193 (85.8)	176 (78.2)	49 (21.8)	38 (16.9)	–	131 (58.2)	5 (2.2)
Erbay, G2 2019	–	190 (90.0)	169 (80.1)	49 (23.2)	24 (11.4)	–	108 (51.2)	6 (2.8)
Santos 2022	9 (13)	49 (71)	13 (19)	15 (22)	13 (19）	11 (16)	–	–
Wienemann 2022	101 (18.6)	398 (73.3)	280 (51.6)	146 (26.9)	82 (15.1)	–	264 (48.6)	–
Echavarría-Pinto 2022	12 (18.7)	55 (84.6)	–	31 (47.7)	–	22 (33.8)	19 (29.2)	–
Diemen 2023	93 (56)	105 (63)	–	35 (21)	86 (52)	87 (52)	157 (93)	–
Xiao 2024	100 (59.5)	–	–	–	40 (23.8)	8 (4.8)	–	–
**Mode=cQFR**								
Tu 2016	–	32 (43.8)	–	17 (27.4)	–	23 (31.5)	28 (38.4)	2 (2.7)
Xu 2017	87 (28.2)	185 (60.1)	139 (45.1)	86 (27.9)	51 (16.6)	48 (15.6)	65 (21.1)	1 (0.3)
Yazaki 2017	33 (23.2)	101 (71.1)	88 (62.0)	41 (28.9)	–	30 (21.2)	58 (40.8)	2 (1.4)
Rosendaelc 2017	3 (18)	11 (65)	–	1 (6)	2 (12)	1 (6)	4 (24)	–
Emori 2018	21 (21)	73 (73)	58 (58)	48 (48)	–	22 (22)	–	–
Stähli 2018	148 (34)	383 (87.8)	345 (79.1)	98 (22.5)	62 (14.2)	143 (32.8)	239 (54.8)	11 (2.5)

Spitaleri 2018	19 (45)	29 (64)	23 (51)	4 (9)	–	2 (4)	2 (4)	0 (0)
Kołtowski 2018	28 (10.4)	203 (75.7)	–	75 (28)	28 (10.4)	128 (47.8)	158 (59)	7 (2.6)
Westra, T 2018	101(59)	121 (70)	–	18 (10)	69 (40)	–	–	–
Mejía-Rentería 2018	56 (23)	164 (66)	143 (58)	94 (38)	46 (19)	–	–	–
Westra, A 2018	156 (57)	201 (74)	186 (68)	78 (29)	73 (27)	–	109 (40)	11(4)
Emori, Prior MI (+) 2018	17 (23)	61 (81)	45 (60)	34 (45)	13 (17)	–	–	–
Emori, Prior MI (–) 2018	23 (31)	64 (85)	46 (61)	36 (48)	12 (16)	–	–	–
Tanigaki 2019	59 (39)	99 (65)	80 (53)	46 (30)	–	–	–	–
Erbay, G1 2019	–	193 (85.8)	176 (78.2)	49 (21.8)	38 (16.9)	–	131 (58.2)	5 (2.2)
Erbay, G2 2019	–	190 (90.0)	169 (80.1)	49 (23.2)	24 (11.4)	–	108 (51.2)	6 (2.8)
Kleczyński 2019	–	–	–	–	–	–	–	–
Smit, DM (+) 2019	–	60 (91)	–	66 (100.0)	–	11 (17)	29 (44)	3 (5)
Smit, DM (–) 2019	–	130 (68)	–	0 (0.0)	–	30 (16)	54 (28)	3 (2)
Kanno 2020	116 (23.0)	352 (69.8)	316 (62.7)	210 (41.7)	–	–	–	–
Mehta 2020	22 (46.8)	27 (57.4)	–	11 (23.4)	–	7 (14.9)	10 (21.3)	–
Tebaldi 2020	34 (29)	89 (77)	66 (57)	31 (27)	–	22 (19)	21 (18)	–
Diemen 2020	79 (47)	83 (49)	–	29 (17)	86 (51)	–	–	–
Gutiérrez-Chico 2020	17 (28.8)	40 (67.8)	31 (52.5)	23 (39.0)	6 (10.2)	28 (47.5)	40 (67.8)	2 (3.4)
Kirigaya 2021	13 (17)	46 (60)	43 (56)	34 (44)	26 (34)	16 (21)	17 (22)	–
Kleczynski 2021	71 (32.1)	197 (89.1)	121 (100.0)	68 (30.8)	–	73 (33.0)	70 (31.7)	–
Diemen 2021	69 (60)	61 (53)	–	18 (16)	49 (44)	29 (25)	55 (48)	–
Xu 2022	17 (35.42)	32 (66.67)	5 (10.42)	12 (25.00)	3 (6.25)	–	11 (22.92)	–
Zasada 2022	–	–	–	–	–	–	–	–
Dowling 2022	16 (46)	24 (69)	23 (66)	20 (57)	–	4 (11)	–	–
Kawashima 2022	122 (63.2)	133 (72.7)	121 (66.1)	66 (36.0)	–	2 (1.1)	–	0 (0)
Wienemann 2022	101 (18.6)	398 (73.3)	280 (51.6)	146 (26.9)	82 (15.1)	–	264 (48.6)	–

Echavarría-Pinto 2022	12 (18.7)	55 (84.6)	–	31 (47.7)	–	22 (33.8)	19 (29.2)	–
Diemen 2023	93 (56)	105 (63)	–	35 (21)	86 (52)	87 (52)	157 (93)	–
Kasinadhuni 2023	8 (15.1)	36 (67.9)	12 (22.6)	21 (39.6)	–	–	–	–
Lopez-Palop 2023	42 (51.9)	58 (71.6)	55 (67.9)	31 (38.3)	–	18 (22.2)	–	–
Xiao 2024	100 (59.5)	–	–	–	40 (23.8)	8 (4.8)	–	–
Yang 2024	40 (51.3)	53 (67.9)	71 (91.0)	31 (39.7)	20 (25.6)	5 (6.4)	19 (24.3)	–
Winter 2024	17 (13)	91 (71)	–	52 (40)	85 (66)	65 (50)	74 (57)	–
**Mode=aQFR**								
Tu 2016	–	32 (43.8)	–	17 (27.4)	–	23 (31.5)	28 (38.4)	2 (2.7)
Rosendaelc 2017	3 (18)	11 (65)	–	1 (6)	2 (12)	1 (6)	4 (24)	–
**Mode=μQFR**								
Tu 2021	–	–	–	–	–	–	–	–
Guan 2022	9 (16.7)	40 (74.0)	29 (53.7)	10 (18.5)	–	4 (7.4)	5 (9.3)	–
Li 2022	71 (42.5)	99 (59.3)	86 (51.5)	49 (29.3)	–	–	–	–
Fezzi 2023	–	102 (82.9)	61 (50.0)	38 (30.9)	–	10 (8.1)	–	–
Kotoku, Distal LM 2023	59 (20.1)	230 (77.2)	207 (70.6)	97 (32.6)	88 (33.1)	10 (3.4)	–	–
Kotoku, pLAD 2023	59 (20.1)	230 (77.2)	207 (70.6)	97 (32.6)	88 (33.1)	10 (3.4)	–	–
Kotoku, pLCX 2023	59 (20.1)	230 (77.2)	207 (70.6)	97 (32.6)	88 (33.1)	10 (3.4)	–	–
Sun 2023	46 (28.22)	91 (55.83)	24 (14.72)	47 (28.83)	4 (2.45)	13 (7.98)	33 (20.25)	3 (1.84)
Zuo, J, Men 2023	147 (48.7)	219 (72.5)	161 (53.3)	92 (30.5)	–	33 (10.9)	90 (29.8)	–
Zuo, J, Women 2023	4 (2.5)	126 (79.7)	53 (33.5)	42 (26.6)	–	7 (4.4)	2 (15.8)	–
Zuo, X, n 2023	150 (32.5)	345 (74.8)	218 (47.3)	131 (28.4)	–	39 (8.5)	110 (23.9)	–
Zuo, X, m 2023	26 (35.6)	57 (78.1)	35 (47.9)	19 (26.0)	–	7 (9.6)	23 (31.5)	–
Yang 2023	72 (27.59)	150 (57.47)	31 (11.88)	74 (28.35)	6 (2.30)	4 (1.53)	52 (19.92)	2 (0.77)
Lai 2024	24 (26.67)	57 (63.33)	9 (10.00)	24 (26.67)	3 (3.33)	3 (3.33)	17 (18.89)	1 (1.11)
Yuta, pre-TAVR 2024	3 (12.0)	22 (88.0)	13 (52.0)	8 (32.0)	–	–	–	–

Yuta, post-TAVR 2024	3 (12.0)	22 (88.0)	13 (52.0)	8 (32.0)	–	–	–	–
**Mode=non-specified QFR**								
Xi 2021	24 (21.8)	44 (40.0)	–	38 (34.5)	3 (2.7)	–	–	–
Gan 2021	11 (21.2)	35 (67.3)	6 (11.5)	13 (25.0)	1 (1.92)	1 (1.92)	12 (23.1)	2 (3.8)
Peper 2021	136 (47.1)	213 (73.7)	–	53 (18.3)	151 (53.0)	–	45 (15.6)	–
Zhang 2022	43 (29.05)	98 (66.22)	99 (66.89)	43 (29.05)	16 (16.15)	–	–	–
Liu 2023	148 (43.92)	224 (66.47)	161 (47.77)	123 (36.50)	–	36 (10.6)	79 (23.44)	–
Dong, DM, 2023	50 (51)	80 (81)	65 (66)	99 (100.0)	26 (26)	7 (7)	21 (21)	0 (0)
Dong, Non-DM, 2023	59 (47)	87 (70)	62 (50)	0 (0)	34 (27)	8 (6)	18 (14)	1 (1)
Yuasa 2023	39 (58.2)	51 (76.1)	–	21 (31.3)	–	20 (29.9)	–	–
Zhang 2024	79 (33.47)	130 (55.08)	78 (33.05)	76 (32.20)	–	–	–	–
Han 2024	28 (30.4)	45 (48.9)	24 (26.1)	30 (32.6)	–	–	–	–
Yuta 2024	3 (12.0)	22 (88.0)	13 (52.0)	8 (32.0)	–	–	–	–
Yuta 2024	3 (12.0)	22 (88.0)	13 (52.0)	8 (32.0)	–	–	–	–

Values are n (%), mean ± standard deviation or median (interquartile range). CAD, coronary artery disease; PCI, percutaneous coronary intervention; CABG, coronary artery bypass grafting. Other abbreviations are as in Supplement 1.

Table S3 Target vessel characteristics in the studies.

Study	No. of vessels	Target vessel, n (%)						Quantitative coronary angiography analysis			
		LAD	D	LCx	OM	RCA	Other	MLD, mm	RVD, mm	%DS, %	LL, mm
**Mode=fQFR**											
Tu 2016	84	46 (54.8)	1 (1.2)	12 (14.3)	5 (6.0)	19 (22.6)	1 (1.2)	1.52 ± 0.36	2.84 (2.57–3.06)	46.1 ± 8.9	–
Rosendaelc 2017	15	12 (80)	0 (0.0)	3 (20)	0 (0.0)	0 (0.0)	0 (0.0)	–	–	38.7 ± 8.6	15.4 ± 7.7
Stähli 2018	516	287 (55.6)	15 (2.9)	67 (13.0)	23 (4.5)	119 (23.1)	5 (1.0)	1.7 (1.4–1.9)	2.8 (2.5–3.2)	41 (36–46)	17.2 (12.0–24.9)
Kołtowski 2018	306	174 (56.9)	220 (71.9)	31 (10.1)	13 (4.2)	81 (26.5)	4 (1.3)	–	–	51.3 ± 10.2	–
Emori, Prior MI (+) 2018	75	48 (64)	0 (0.0)	5 (7)	0 (0.0)	22 (29)	0 (0.0)	1.27 ± 0.06	2.71 ± 0.06	53 ± 14	20.2 ± 9.6
Emori, Prior MI (–) 2018	75	49 (65)	0 (0.0)	12 (16)	0 (0.0)	14 (19)	0 (0.0)	1.30 ± 0.06	2.79 ± 0.06	54 ± 14	20.2 ± 9.9
Erbay, G1 2019	270	157 (58.1)	12 (4.5)	44 (16.3)	17 (6.3)	35 (13.0)	5 (1.8)	1.4 (1.3–1.6)	2.5 (2.3–2.7)	41.4 (36.4–47.6)	17.0 (11.7–24.0)
Erbay, G2 2019	246	130 (52.8)	3 (1.2)	23 (9.4)	6 (2.5)	84 (34.1)	0 (0.0)	1.9 (1.7–2.1)	3.3 (3.0–3.6)	41.4 (36.4–45.7)	17.2 (12.1–25.9)
Santos 2022	75	43 (57.4)	2 (2.7)	6 (8)	1 (1.3)	22 (29.3)	1 (1.3)	1.31 ± 0.32	2.43 ± 0.49	46.2 ± 7.4	18.95 ± 12.97
Wienemann 2022	626	385 (61.5)	0 (0.0)	103 (16.5)	0 (0.0)	126 (20.1)	12 (1.9)	1.5 (1.3–1.8)	–	44.5 ± 7.5	18.5 (12.3–26.1)
Echavarría-Pinto 2022	90	48 (53.3)	2 (2.2)	19 (21.1)	4 (4.4)	13 (14.4)	4 (4.4)	1.5 ± 0.5	2.9 ± 0.6	46.6 ± 12.8	17.2 (10.9–30.5)
Diemen 2023	334	127 (38)	0 (0.0)	114 (34)	0 (0.0)	93 (28)	0 (0.0)	1.7 ± 0.5	–	41 ± 14	16.3 (10.5–26.2)
Xiao 2024	246	169 (68.7)	0 (0.0)	35 (14.2)	0 (0.0)	42 (17.1)	0 (0.0)	–	–	–	–
**Mode=cQFR**											
Tu 2016	84	46 (54.8)	1 (1.2)	12 (14.3)	5 (6.0)	19 (22.6)	1 (1.2)	1.52 ± 0.36	2.84 (2.57–3.06)	46.1 ± 8.9	–
Xu 2017	332	185 (55.7)	2 (0.6)	49 (14.8)	5 (1.5)	87 (26.2)	4 (1.2)	1.51 ± 0.44	2.82 ± 0.56	46.5 ± 11.3	13.1 ± 6.4
Yazaki 2017	151	96 (63.6)	2 (1.3)	25 (16.6)	0 (0.0)	26 (17.2)	2 (1.3)	1.38 ± 0.39	2.84 ± 0.57	48.8 ± 8.2	16.8 (12.1–24.6)
Rosendaelc 2017	15	12 (80)	0 (0.0)	3 (20)	0 (0.0)	0 (0.0)	0 (0.0)	–	–	38.7 ± 8.6	15.4 ± 7.7
Emori 2018	100	63 (63)	0 (0.0)	23 (23)	0 (0.0)	14 (14)	0 (0.0)	1.19 ± 0.39	2.62 ± 0.55	55 ± 10	22.6 ± 12.4
Stähli 2018	516	287 (55.6)	15 (2.9)	67 (13.0)	23 (4.5)	119 (23.1)	5 (1.0)	1.7 (1.4–1.9)	2.8 (2.5–3.2)	41 (36–46)	17.2 (12.0–24.9)

Spitaleri 2018	49	–	–	–	–	–	–	–	3 ± 0.4	66 ± 10	–
Kołtowski 2018	306	174 (56.9)	220 (71.9)	31 (10.1)	13 (4.2)	81 (26.5)	4 (1.3)	–	–	51.3 ± 10.2	–
Westra, T 2018	255	129 (51)	17 (7)	29 (11)	23 (9)	46 (18)	11 (4)	–	–	–	–
Mejía-Rentería 2018	300	177 (59.0)	13 (4.3)	37 (12.3)	18 (6.0)	49 (16.3)	6 (2.0)	1.30 (1.00–1.60)	2.80 ± 0.57	52 ± 12	75.9 ± 22.4
Westra, A 2018	317	160 (50)	5 (1)	50 (16)	23 (7)	68 (22)	11 (3)	1.57 (1.27–1.90)	2.82 (2.44–3.20)	45 ± 10	9.64 (7.53–13.76)
Emori, Prior MI (+) 2018	75	48 (64)	0 (0.0)	5 (7)	0 (0.0)	22 (29)	0 (0.0)	1.27 ± 0.06	2.71 ± 0.06	53 ± 14	20.2 ± 9.6
Emori, Prior MI (–) 2018	75	49 (65)	0 (0.0)	12 (16)	0 (0.0)	14 (19)	0 (0.0)	1.30 ± 0.06	2.79 ± 0.06	54 ± 14	20.2 ± 9.9
Tanigaki 2019	233	132 (56)	0 (0.0)	53 (23)	0 (0.0)	48 (21)	0 (0.0)	1.38 ± 0.46	2.87 ± 0.65	49 ± 13	20.4 ± 9.8
Erbay, G1 2019	270	157 (58.1)	12 (4.5)	44 (16.3)	17 (6.3)	35 (13.0)	5 (1.8)	1.4 (1.3–1.6)	2.5 (2.3–2.7)	41.4 (36.4–47.6)	17.0 (11.7–24.0)
Erbay, G2 2019	246	130 (52.8)	3 (1.2)	23 (9.4)	6 (2.5)	84 (34.1)	0 (0.0)	1.9 (1.7–2.1)	3.3 (3.0–3.6)	41.4 (36.4–45.7)	17.2 (12.1–25.9)
Kleczyński 2019	123	48 (39.0)	–	–	–	–	75 (61.0)	–	–	44.2 ± 11.7	–
Smit, DM (+) 2019	82	55 (67)	0 (0.0)	13 (16)	0 (0.0)	9 (11)	5 (6)	1.6 ± 0.3	–	42.7 ± 8.9	20.8 (11.9–31.8)
Smit, DM (–) 2019	238	161 (68)	0 (0.0)	33 (14)	0 (0.0)	24 (10)	20 (8)	1.6 ± 0.3	–	43.3 ± 8.5	20.0 (12.7–28.6)
Kanno 2020	504	348 (69.0)	0 (0.0)	56 (11.1)	0 (0.0)	100 (19.8)	0 (0.0)	1.4 (1.1–1.7)	2.8 (2.5–3.1)	49.6 ± 12.0	18.4 (13.1–24.0)
Mehta 2020	85	38 (44.7)	0 (0.0)	15 (17.6)	10 (11.8)	17 (20.0)	5 (5.9)	–	–	35.3 ± 14.7	11.9 ± 10.2
Tebaldi 2020	184	100 (54)	0 (0.0)	44 (24)	0 (0.0)	40 (22)	0 (0.0)	–	2.7 (2.5–3.4)	62 (55–75)	21 (13–25)
Diemen 2020	286	122 (43)	3 (1)	58 (20)	12 (4)	77 (27)	14 (4.9)	1.7 ± 0.6	–	37 ± 16	18 ± 13
Gutiérrez-Chico 2020	75	39 (52.0)	5 (6.7)	4 (5.3)	2 (2.7)	23 (30.7)	2 (2.7)	–	–	–	–
Kirigaya 2021	95	61 (64)	0 (0.0)	17 (18)	0 (0.0)	17 (18)	0 (0.0)	1.42 ± 0.38	2.82 ± 0.61	49.2 ± 8.6	20.3 ± 10.8
Kleczynski 2021	416	190 (45.7)	19 (4.6)	64 (15.4)	23 (5.5)	88 (21.2)	32 (7.7)	1.5 ± 0.5	3.4 ± 0.6	58.6 ± 13.4	18.5 ± 9.9
Diemen 2021	134	70 (52)	2 (1)	14 (10)	8 (6)	33 (25)	7 (5.2)	0.98 ± 0.36	–	64 ± 12	–
Xu 2022	48	26 (54.17)	0 (0.0)	5 (10.42)	0 (0.0)	17 (35.42)	0 (0.0)	–	–	47.2 ± 11.2	–
Zasada 2022	13	12 (92)	0 (0.0)	1 (7.7)	0 (0.0)	0 (0.0)	0 (0.0)	–	–	–	–
Dowling 2022	57	31 (54)	5 (9)	5 (9)	9 (16)	3 (5)	4 (7)	–	–	–	–
Kawashima 2022	469	190 (40.5)	0 (0.0)	143 (30.5)	0 (0.0)	136 (29.0)	0 (0.0)	–	2.75 ± 0.64	–	–

Wienemann 2022	626	385 (61.5)	0 (0.0)	103 (16.5)	0 (0.0)	126 (20.1)	12 (1.9)	1.5 (1.3–1.8)	–	44.5 ± 7.5	18.5 (12.3–26.1)
Echavarría-Pinto 2022	90	48 (53.3)	2 (2.2)	19 (21.1)	4 (4.4)	13 (14.4)	–	1.5 ± 0.5	2.9 ± 0.6	46.6 ± 12.8	17.2 (10.9–30.5)
Diemen 2023	334	127 (38)	0 (0.0)	114 (34)	0 (0.0)	93 (28)	0 (0.0)	1.7 ± 0.5	–	41 ± 14	16.3 (10.5–26.2)
Kasinadhuni 2023	56	28 (50)	0 (0.0)	15 (26.8)	0 (0.0)	12 (21.5)	1 (1.7)	1.35 ± 0.33	3.2 ± 0.37	45.25 ± 11.22	20.8 ± 12.4
Lopez-Palop 2023	107	82 (76.6)	0 (0.0)	25 (23.4)	0 (0.0)	0 (0.0)	0 (0.0)	1.86 ± 0.40	3.59 ± 1.67	45.88 ± 8.80	13.35 ± 6.92
Xiao 2024	246	169 (68.7)	0 (0.0)	35 (14.2)	0 (0.0)	42 (17.1)	0 (0.0)	–	–	–	–
Yang 2024	98	67 (68.4)	0 (0.0)	22 (22.4)	0 (0.0)	3 (3.1)	6 (6.1)	1.82 ± 0.34	3.85 ± 0.65	52.1 ± 8.5	20.12 ± 14.33
Winter 2024	140	43 (31)	–	89 (64)	–	64 (46)	–	1.77 ± 0.70	3.04 ± 0.71	42.26 ± 15.56	10.59 (6.41–17.61)
**Mode=aQFR**											
Tu 2016	84	46 (54.8)	1 (1.2)	12 (14.3)	5 (6.0)	19 (22.6)	1 (1.2)	1.52 ± 0.36	2.84 (2.57–3.06)	46.1 ± 8.9	–
Rosendaelc 2017	15	12 (80)	0 (0.0)	3 (20)	0 (0.0)	0 (0.0)	0 (0.0)	–	–	38.7 ± 8.6	15.4 ± 7.7
**Mode=μQFR**											
Tu 2021	330	–	–	–	–	–	–	–	–	–	–
Guan 2022	61	41 (67.2)	0 (0.0)	9 (14.8)	0 (0.0)	11 (18.0)	0 (0.0)	–	–	–	–
Li 2022	191	131 (68.6)	7 (3.7)	24 (12.6)	2 (1.0)	27 (14.1)	0 (0.0)	1.60 ± 0.40	–	40.2 ± 8.2	–
Fezzi 2023	198	116 (58.6)	–	–	–	38 (19.2)	–	1.69 ± 0.69	2.80 ± 0.57	42.9 ± 16.4	14.8 ± 7.0
Kotoku, Distal LM 2023	300	–	–	–	–	–	–	–	4.06 ± 0.82	–	–
Kotoku, pLAD 2023	300	–	–	–	–	–	–	–	2.78 ± 0.71	–	–
Kotoku, pLCX 2023	300	–	–	–	–	–	–	–	2.63 ± 0.62	–	–
Sun 2023	178	123 (69.10)	2 (1.12)	24 (13.48)	0 (0.0)	29 (16.29)	0 (0.0)	–	3.14 ± 0.70	47.38 ± 12.30	–
Zuo, J, Men 2023	329	220 (66.9)	0 (0.0)	56 (17.0)	0 (0.0)	53 (16.1)	0 (0.0)	1.80 (1.54–2.16)	3.0 (2.6–3.5)	39.4 ± 8.1	21.6 (11.8–33.1)
Zuo, J, Women 2023	168	115 (68.5)	0 (0.0)	23 (13.7)	0 (0.0)	30 (17.9)	0 (0.0)	1.87 (1.57–2.19)	2.9 (2.5–3.4)	36.4 ± 8.2	18.3 (11.6–27.3)
Zuo, X, n 2023	496	331 (66.7)	0 (0.0)	84 (16.9)	0 (0.0)	81 (16.3)	0 (0.0)	1.81 (1.54–2.17)	–	37.8 ± 8.4	19.3 (11.7–30.2)
Zuo, X, m 2023	75	56 (74.7)	0 (0.0)	7 (9.3)	0 (0.0)	12 (16.0)	0 (0.0)	1.77 (1.52–2.10)	–	39.8 ± 7.3	24.9 (16.7–34.4)

Yang 2023	286	194 (67.83)	2 (0.70)	40 (13.99)	1 (0.35)	49 (17.13)	0 (0.0)	–	3.19 ± 0.70	47.31 ± 12.21	–
Lai 2024	90	58 (64.44)	0 (0.0)	12 (13.33)	0 (0.0)	20 (22.22)	0 (0.0)	–	3.25 ± 0.78	50.08 ± 8.95	–
Yuta, pre-TAVR 2024	38	21 (55.3)	0 (0.0)	10 (26.3)	0 (0.0)	7 (18.4)	0 (0.0)	1.82 ± 0.60	–	33.8 ± 13.0	11.8 ± 6.8
Yuta, post-TAVR 2024	38	21 (55.3)	0 (0.0)	10 (26.3)	0 (0.0)	7 (18.4)	0 (0.0)	1.96 ± 0.70	–	31.3 ± 13.6	11.2 ± 7.1
**Mode=non-specified QFR**											
Xi 2021	110	–	–	–	–	–	–	–	–	–	–
Gan 2021	52	35 (67.3)	0 (0.0)	8 (15.4)	0 (0.0)	9 (17.3)	0 (0.0)	–	3.20 ± 0.62	48.97 ± 10.15	–
Peper 2021	381	117 (30.7)	13 (3.4)	35 (9.2)	0 (0.0)	195 (51.2)	21 (5.5)	1.35 ± 0.38	2.63 (0.57)	48.5 ± 9.4	20.3 (12.7–24.1)
Zhang 2022	175	119 (68)	0 (0.0)	29 (16.57)	0 (0.0)	27 (15.43)	0 (0.0)	1.37 ± 0.46	2.66 ± 0.60	48.29 ± 13.2	10.52 ± 4.55
Liu 2023	337	264 (78.34)	0 (0.0)	23 (6.82)	0 (0.0)	50 (14.84)	0 (0.0)	1.60 (1.39–1.84)	2.80 (2.50–3.20)	43.00 (37.00–49.00)	26.90 (16.15–39.75)
Dong, DM, 2023	142	75 (53)	0 (0.0)	21 (15)	0 (0.0)	29 (20)	17 (12)	1.4 (1.1–1.6)	–	44 ± 9	26.0 (16.1–38.3)
Dong, Non-DM, 2023	175	105 (60)	0 (0.0)	27 (15)	0 (0.0)	29 (17)	14 (8)	1.5 (1.2–1.7)	–	45 ± 10	26.4 (16.9–39.9)
Yuasa 2023	73	64 (87.7)	1 (1.4)	7 (9.6)	1 (1.4)	0 (0.0)	0 (0.0)	1.82 ± 0.39	3.38 ± 0.88	43.81 ± 10.84	–
Zhang 2024	236	186 (78.81)	0 (0.0)	13 (5.51)	0 (0.0)	37 (15.68)	0 (0.0)	–	–	–	–
Han 2024	103	54 (52.4)	0 (0.0)	13 (12.6)	0 (0.0)	36 (35.0)	0 (0.0)	–	–	–	–
Yuta 2024	38	21 (55.3)	0 (0.0)	10 (26.3)	0 (0.0)	7 (18.4)	0 (0.0)	1.82 ± 0.60	–	33.8 ± 13.0	11.8 ± 6.8
Yuta 2024	38	21 (55.3)	0 (0.0)	10 (26.3)	0 (0.0)	7 (18.4)	0 (0.0)	1.96 ± 0.70	–	31.3 ± 13.6	11.2 ± 7.1

Values are n (%), mean ± standard deviation or median (interquartile range). LAD, left descending artery; D, diagonal; LCx, left circumflex; OM, obtuse marginal; RCA, right coronary artery; MLD, minimum lumen diameter; RVD, reference vessel diameter; DS, diameter stenosis; LL, lesion length. Other abbreviations are as in Supplement 1.

Table S4 Individual study estimates of per-vessel diagnostic accuracy of QFR.

Study	Accuracy, %	Sensitivity, %	Specificity, %	PPV, %	NPV, %	LR+	LR−	AUC
**Mode=fQFR**								
Tu 2016	80 (71–89)	67 (46–84)	86 (74–94)	69 (48–86)	85 (73–93)	4.8 (2.4–9.5)	0.4 (0.2–0.7)	0.88 (0.79–0.94)
Rosendaelc 2017	86.67	100.00	84.62	50.00	100.00	6.50	0.00	–
Stähli 2018	91.9 (89.2–94.1)	72.0 (62.1–80.5)	96.6 (94.4–98.2)	83.7 (75.2–89.7)	93.5 (91.3–95.2)	21.4 (12.6–36.3)	0.29 (0.21–0.40)	0.84 (0.81–0.87)
Kołtowski 2018	74.5 (68.7–78.1)	89.5	63.4	64.4	89.1	2.4	0.2	0.87 (0.83–0.91)
Emori, Prior MI (+) 2018	77 (68–81)	94 (85–98)	62 (53–65)	69 (62–72)	92 (79–98)	2.5 (1.8–2.8)	0.1 (0.0–0.3)	0.90 (0.81–0.95)
Emori, Prior MI (–) 2018	87 (78–89)	98 (90–99)	73 (63–75)	82 (76–84)	96 (84–99)	3.6 (2.5–4.0)	0.0 (0.0–0.2)	0.97 (0.93–0.99)
Erbay, G1 2019	91.1 (87.1–94.2)	73.9 (61.5–84.0)	96.6 (93.1–98.6)	87.3 (76.6–93.5)	92.1 (88.6–94.6)	21.6 (10.9–45.4)	0.27 (0.2–0.4)	0.98 (0.96–0.99)
Erbay, G2 2019	92.7 (88.7–95.6)	68.6 (50.7–83.2)	96.7 (93.3–98.7)	77.4 (61.5–88.0)	94.9 (91.9–96.8)	20.7 (9.7–44.3)	0.3 (0.2–0.5)	0.97 (0.94–0.99)
Santos 2022	84.0 (75.6–92.4)	67.9 (47.6–84.1)	93.6 (82.5–98.7)	86.4 (65.2–95.5)	83.0 (70.4–90.9)	10.29 (1.97–28.4)	0.34 (0.23–0.53)	0.85 (0.75–0.92)
Wienemann 2022	83 (80.0–86.1)	60 (52.4–66.7)	94 (91.0–95.8)	80 (72.8–86.5)	84 (80.5–87.2)	9.27 (6.36–13.52)	0.43 (0.36–0.51)	0.891 (0.865–0.918)
Echavarría-Pinto 2022	82.2	83.3	80.6	75.00	88.00	4.50	0.20	0.90 (0.86–0.97)
Diemen 2023	84 (79–88)	68 (56–78)	88 (84–92)	61 (50–71)	91 (87–94)	5.75	0.36	–
Xiao 2024	76.83	59	89.04	78.67	76.02	5.38	0.46	0.821 (0.766-0.875)
**Mode=cQFR**								
Tu 2016	86 (78–93)	74 (54–89)	91 (81–97)	80 (59–93)	88 (77–95)	8.4 (3.6–20.1)	0.3 (0.1–0.5)	0.92 (0.85–0.97)
Xu 2017	92.7 (89.3–95.3)	94.6 (88.7–98.0)	91.7 (87.1–95.0)	85.5 (78.0–91.2)	97.1 (93.7–98.9)	11.4 (7.1–17.0)	0.06 (0.03–0.13)	0.96 (0.94–0.98)

Yazaki 2017	88.7	89.1	88.6	77.4	94.9	7.80	0.12	0.93
Rosendaelc 2017	86.67	100.00	84.62	50.00	100.00	6.50	0.00	–
Emori 2018	94 (88–97)	97 (92–99)	87 (77–92)	94 (90–96)	93 (82–98)	7.5 (4.0–11.7)	0.0 (0.0–0.1)	–
Stähli 2018	93.4 (90.9–95.4)	75.0 (65.3–83.1)	97.8 (95.9–99.0)	89.3 (81.2–94.1)	94.2 (92.1–95.8)	34.7 (18.0–66.8)	0.26 (0.18–0.36)	0.86 (0.83–0.89)
Spitaleri 2018	94	88	97	94	94	28.88	0.13	0.96 (0.89–0.99)
Kołtowski 2018	85.4 (78.7–89.5)	83.8	86.6	82.2	87.9	6.3	0.2	0.94 (0.91–0.97)
Westra, T 2018	83	77 (66–85)	86 (79–91)	75 (65–84)	87 (80–92)	5.4 (3.6–8)	0.27 (0.18–0.4)	0.86 (0.81–0.91)
Mejía-Rentería 2018	88	89 (83–94)	87 (80–91)	85 (79–89)	91 (86–94)	6.6 (4.5–9.8)	0.1 (0.1–0.2)	0.93 (0.90–0.96)
Westra, A 2018	86.8	86.5 (78.4–92.4)	86.9 (81.6–91.1)	76.3 (67.6–83.6)	93.0 (88.5–96.1)	6.58 (4.62–9.37)	0.16 (0.09–0.25)	0.92 (0.89–0.96)
Emori, Prior MI (+) 2018	87 (77–92)	92 (82–97)	82 (73–87)	83 (74–87)	91 (82–97)	5.1 (3.0–7.3)	0.1 (0.0–0.2)	0.93 (0.86–0.97)
Emori, Prior MI (–) 2018	92 (84–96)	95 (88–99)	88 (79–92)	91 (84–94)	94 (84–98)	7.9 (4.1–12.4)	0.1 (0.0–0.2)	0.97 (0.93– 0.99)
Tanigaki 2019	85 (81–89)	90 (85–94)	82 (77–85)	81 (76–84)	90 (85–94)	4.9 (3.7–6.2)	0.1 (0.1–0.2)	0.93
Erbay, G1 2019	94.1 (90.6–96.6)	80.0 (68.2–88.9)	98.5(95.8–99.7)	94.6 (84.9–98.2)	94.0 (90.5–96.2)	54.7 (17.7–169.2)	0.2 (0.1–0.3)	0.98 (0.96–0.99)
Erbay, G2 2019	92.7 (88.7–95.6)	65.7 (47.8–80.9)	97.2 (93.9–99.0)	79.3 (62.7–89.7)	94.5 (91.5–96.4)	23.1 (10.1–52.7)	0.35 (0.2–0.6)	0.97 (0.94–0.99)
Kleczyński 2019	95.1	91.8	97.3	95.74	94.74	33.98	0.08	0.98 (0.94–1.00)
Smit, DM (+) 2019	88 (79–94)	71 (49–87)	95 (86–99)	85 (65–95)	89 (81–94)	13.69	0.31	0.91 (0.84–0.99)
Smit, DM (–) 2019	85 (79–89)	69 (56–79)	91 (85–95)	74 (64–83)	88 (84–91)	7.83	0.34	0.93 (0.89–0.96)
Kanno 2020	78.6	85.3	72.6	73.6	84.6	3.11	0.20	0.84 (0.81–0.88)
Mehta 2020	93	86	95	86	95	18.29	0.15	–
Tebaldi 2020	88	72	94	82	90	11.80	0.30	0.964 (0.903–0.974)
Diemen 2020	88 (84–92)	70 (57–81)	93 (89–96)	73 (62–82)	92 (89–94)	9.91	0.32	0.94 (0.91–0.97)

Gutiérrez-Chico 2020	90 (83–97)	91 (72–99)	89 (77–97)	81 (61–93)	96 (85–99)	8.6 (3.7–19.8)	0.1 (0.0–0.4)	0.91
Kirigaya 2021	85.20	80.40	91.00	91.10	80.00	8.84	0.22	–
Kleczynski 2021	94.2 (91.5–96.3)	100.0 (96.6–100.0)	92.2 (88.6–94.9)	81.8 (75.4–86.9)	100	12.83	0.00	–
Diemen 2021	94 (88–97)	100 (97–100)	27 (6–61)	94 (91–95)	100	1.38	0.00	–
Xu 2022	81.3 (71.3–91.2)	75.0 (62.3–92.3)	84.4 (73.0–95.2)	70.6 (62.3–96.6)	87.1 (75.2–94.7)	4.808	0.208	0.862 (0.824–0.931)
Zasada 2022	61.54	40.00	75.00	50.00	66.67	1.60	0.80	–
Dowling 2022	84	73	91	84	84	8.48	0.30	0.92 (0.84–1.00)
Kawashima 2022	88.27	90.10	78.67	95.69	60.20	4.22	0.13	–
Wienemann 2022	91 (88.2–92.9)	82 (75.5–86.9)	95 (92.2–96.6)	87 (81.3–91.7)	92 (89.3–94.5)	15.45 (10.3–23.1)	0.19 (0.14–0.26)	0.938 (0.917–0.961)
Echavarría-Pinto 2022	83.3	85.2	80.6	75.61	89.80	4.65	0.17	0.92 (0.86–0.97)
Diemen 2023	84 (80–88)	72 (61–81)	87 (83–91)	61 (50–71)	92 (88–95)	5.57	0.32	–
Kasinadhuni 2023	92.80	87.50	95.00	87.50	95.00	17.50	0.13	0.97 (0.94–1.00)
Lopez-Palop 2023	90.7 (83.5–95.4)	88.1 (75.0–94.8)	92.3 (83.2–96.7)	88.1 (77.1–99.1)	92.3 (85.1–99.5)	11.5 (4.9–26.8)	0.13 (0.1–0.3)	0.93 (0.88–0.98)
Xiao 2024	81.30	56.00	98.63	96.55	76.60	40.88	0.45	0.829 (0.773-0.885)
Yang 2024	90 (84–94)	85 (74–92)	95 (88–99)	93 (84–97)	88 (91–93)	16.7 (6.4–43.7)	0.16 (0.09–0.29)	0.93 (0.87–0.96)
Winter 2024	83 (76–88)	84 (70–92)	82 (74–89)	68 (55–79)	92 (84–96)	4.78	0.20	0.90 (0.84–0.95)
**Mode=aQFR**								
Tu 2016	87 (80-94)	78 (58-91)	91 (81-97)	81 (61-93)	90 (79-96)	8.9 (3.7-21.0)	0.2 (0.1-0.5)	0.91 (0.83-0.96)
Rosendaelc 2017	86.67	50.00	92.31	50.00	92.31	6.50	0.54	–
**Mode=μQFR**								

Tu 2021	93.0 (90.2–95.8)	87.5 (80.2–92.8)	96.2 (92.6–98.3)	92.9 (86.5–96.9)	93.1 (88.9–96.1)	23.0 (11.6–45.5)	0.13 (0.08–0.20)	0.97 (0.95–0.99)
Guan 2022	96.7 (88.7–99.6)	100.0 (92.0–100.0)	88.2 (63.6–98.5)	95.7 (85.7–98.8)	100.0 (100–100.0)	8.50	0.00	–
Li 2022	93 (90–97)	91 (83–97)	95 (89–98)	93 (84–97)	94 (87–97)	16.8 (7.7–36.6)	0.09 (0.04–0.2)	0.97 (0.93–0.99)
Fezzi 2023	93.4 (89.9–96.9)	82.5 (67.2–92.7)	96.8 (92.8–99.0)	86.8 (71.9–95.6)	95.6 (91.2–98.2)	26.07(10.9–62.5)	0.18 (0.09–0.4）	0.930 (0.875–0.965)
Kotoku, Distal LM 2023	98.3 (96.2–99.5)	81.2 (54.4–96.0)	99.3 (97.5–99.9)	86.7 (59.5–98.3)	98.9 (97.0–99.8)	115.38 (28.43–468.31)	0.19 (0.07–0.52)	0.95 (0.87–1.00)
Kotoku, pLAD 2023	95.3 (92.3–97.4)	88.2 (76.1–95.6)	96.8 (93.8–98.6)	84.9 (72.4–93.3)	97.6 (94.8–99.1)	27.46 (13.79–54.70)	0.12 (0.06–0.26)	0.94 (0.89–0.99)
Kotoku, pLCX 2023	95.3 (92.3–97.4)	84.8 (71.1–93.7)	97.2 (94.4–98.9)	84.8 (71.1–93.7)	97.2 (94.4–98.9)	30.76 (14.67–64.53)	0.16 (0.08–0.31)	0.94 (0.89–0.99)
Sun 2023	95.51 (91.34–98.04)	85.45 (73.34–95.51)	100.00 (97.05–100.00)	100.00	93.89 (89.01–96.69)	–	0.15	0.96 (0.93–0.99)
Zuo, J, Men 2023	83.3 (78.8–87.2)	66.0 (56.0–75.1)	91.2 (86.7–94.5)	77.3 (68.6–84.1)	85.5 (81.8–88.5)	7.5 (4.8–11.6)	0.4 (0.3–0.5)	–
Zuo, J, Women 2023	92.9 (87.9–96.3)	74.1 (53.7–88.9)	96.5 (91.9–98.8)	80.0 (62.2–90.7)	95.1 (91.1–97.4)	20.9 (8.6–50.8)	0.3 (0.1–0.5)	–
Zuo, X, n 2023	88 (85–91)	70 (61–78)	94 (91–96)	77 (69–83)	91 (89–93)	11.66	0.32	0.91 (0.88–0.93)
Zuo, X, m 2023	80 (69–88)	69 (52–84)	90 (76–97)	86 (71–94)	76 (66–84)	6.77	0.34	0.87 (0.78–0.94)
Yang 2023	94.06 (90.65–96.50)	82.56 (72.87–89.90)	99.00 (96.44–99.88)	97.26 (89.91–99.30)	92.96 (89.29–95.44)	82.56 (20.72–328.95)	0.18 (0.11–0.28)	0.96
Lai 2024	95.56 (89.01–98.78)	90.91 (75.67–98.09)	98.25 (90.61–99.96)	96.77 (81.04–99.53)	94.92 (86.38–98.21)	51.82 (7.40–362.66)	0.09 (0.03–0.27)	–
Yuta, pre-TAVR 2024	84.2 (68.7–93.4)	61.6 (31.6–86.1)	96.0 (79.6–99.9)	88.9 (52.8–98.3)	82.8 (70.6–90.6)	15.38	0.40	0.91 (0.77–0.98)
Yuta, post-TAVR 2024	86.1 (70.5–93.7)	66.7 (34.9–90.1)	95.8 (78.9–99.9)	88.9 (53.0–98.3)	85.2 (72.0–92.8)	17.31	0.32	0.93 (0.79–0.99)

**Mode=non-specified**								
**QFR**								
Xi 2021	83.6 (75.5–89.5)	75.0 (61.7–84.9)	91.4 (81.0–96.7)	88.6 (75.6–95.5)	80.3 (69.0–88.3)	8.70	0.27	0.94
Gan 2021	83.3 (72.2–92.6)	85.7 (66.7–100.0)	87.5 (75.0–96.9)	81.8 (63.8–95.5)	90.0 (80.0–100.0)	6.856	0.163	–
Peper 2021	85.6	84.6	96.3	83.1	87.6	6.19	0.18	0.89
Zhang 2022	82.29	80.25	84.04	81.25	83.16	5.03	0.24	0.86 (0.80–0.91)
Liu 2023	81.01	77.78	83.70	79.87	81.91	4.77	0.27	0.823 (0.775–0.870)
Dong, DM, 2023	90 (84–94)	70 (56–81)	90 (81–95)	81 (69–89)	82 (75–87)	6.7 (3.5–12.7)	0.3 (0.2–0.5)	0.90 (0.84–0.94)
Dong, Non-DM, 2023	92 (87–96)	85 (74–92)	89 (82–94)	82 (73–89)	91 (85–95)	7.8 (4.8–13.4)	0.2 (0.1–0.3)	0.92 (0.87–0.96)
Yuasa 2023	79.4	85.4	69.6	85.4	64.0	2.83	0.20	–
Zhang 2024	78.0	60.9	92.9	88.2	73.1	8.53	0.42	–
Han 2024	92.2 (85.3–96.6)	87.2 (74.3–95.2)	96.4 (87.7–99.6)	95.4 (84.0–98.8)	90.0 (81.0–95.0)	24.4 (6.2–95.7)	0.13 (0.06–0.28)	0.987 (0.842–0.999)
Yuta 2024	81.5 (65.7–92.3)	69.2 (38.6–90.9)	88.0 (68.8–97.5)	75.0 (49.4–90.2)	84.6 (70.6–92.6)	5.77	0.35	0.91 (0.77–0.98)
Yuta 2024	81.5 (61.9–93.7)	55.6 (21.2–86.3)	94.4 (72.7–99.9)	83.3 (40.5–97.3)	81.0 (67.0–89.9)	13.46	0.48	0.89 (0.71–0.98)

Values are mean (95% confidence interval). PPV, positive predictive value; NPV, negative predictive value; LR+, positive likelihood ratio; LR−, negative likelihood ratio; AUC, area under the receiver operating characteristic curve. Other abbreviations are as in Supplement 1.

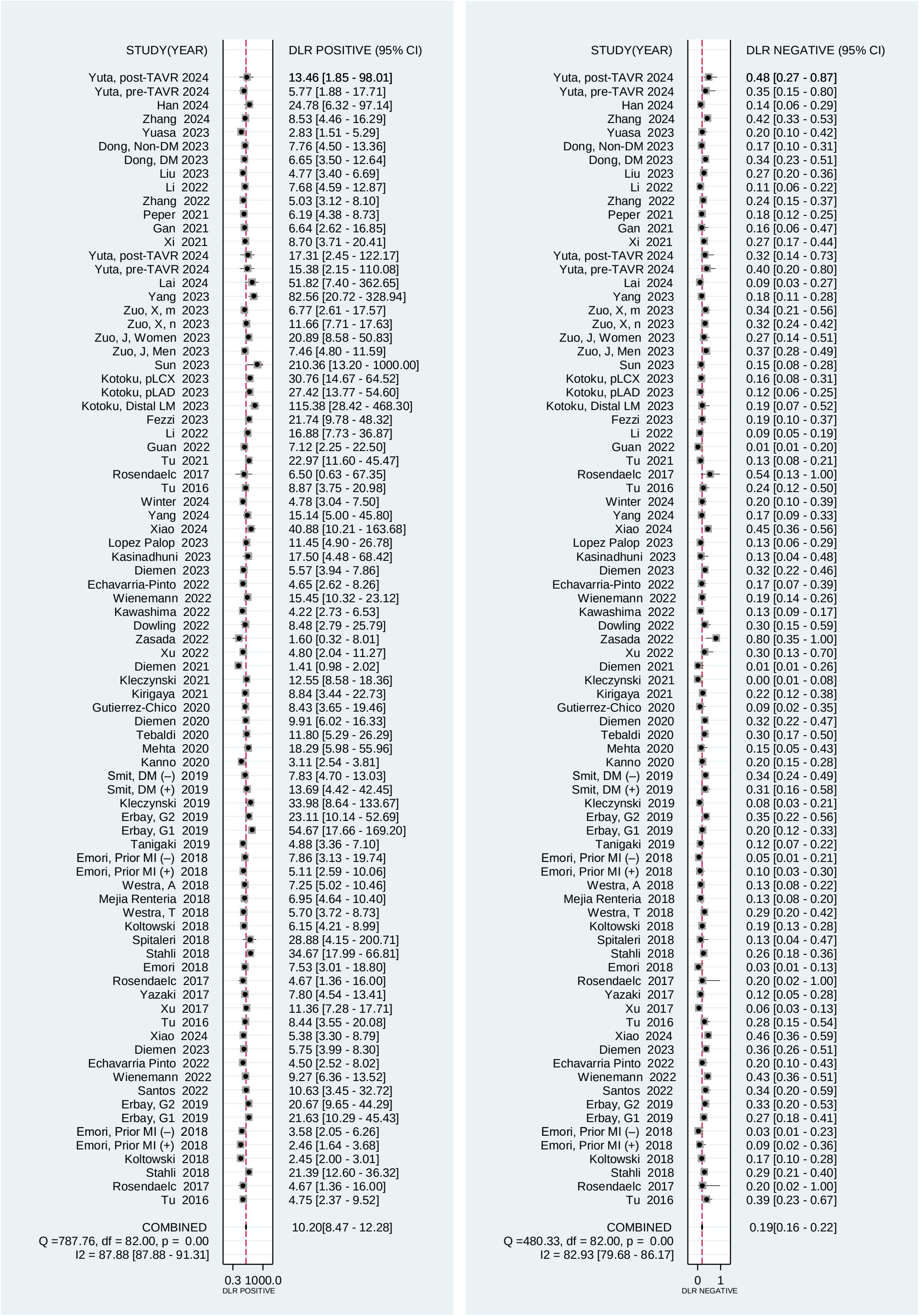

Figure S1 Forest plots of LR+ and LR- of QFR.

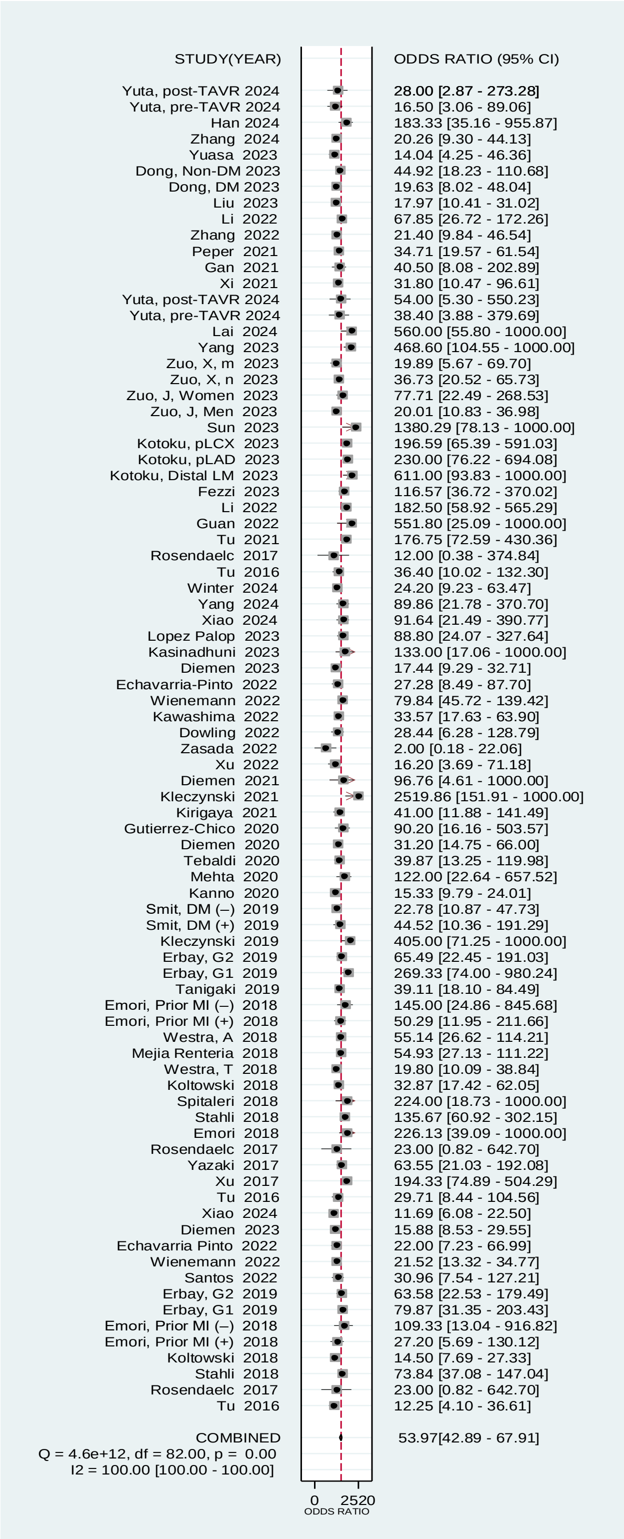

Figure S2 Forest plots of DOR of QFR.

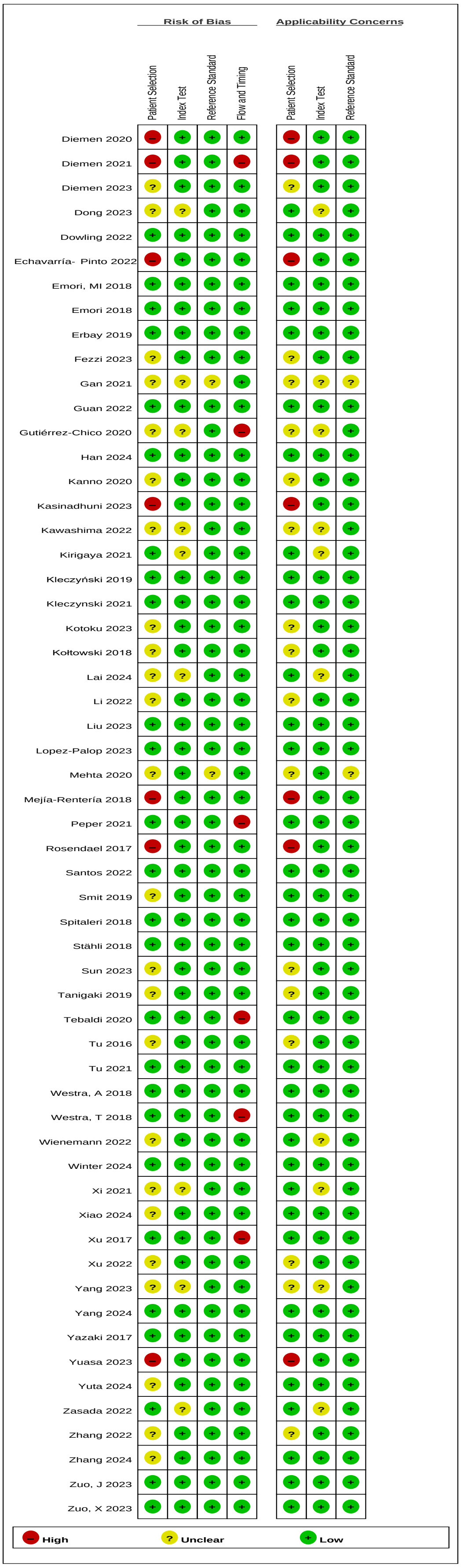

Figure S3 Quality assessment of the included studies.
